# Commute Booster: A Mobile Application for First/Last Mile and Middle Mile Navigation Support for People With Blindness and Low Vision

**DOI:** 10.1109/JTEHM.2023.3293450

**Published:** 2023-07-07

**Authors:** Junchi Feng, Mahya Beheshti, Mira Philipson, Yuvraj Ramsaywack, Maurizio Porfiri, John-Ross Rizzo

**Affiliations:** Department of Biomedical EngineeringTandon School of EngineeringNew York University 5894 Brooklyn NY 11201 USA; Center for Urban Science and Progress, Tandon School of EngineeringNew York University 5894 Brooklyn NY 11201 USA; Department of Mechanical and Aerospace EngineeringTandon School of EngineeringNew York University 5894 Brooklyn NY 11201 USA; Department of Rehabilitation MedicineNYU Langone Health 12297 New York NY 10016 USA; Metropolitan Transportation Authority 51983 New York NY 10004 USA

**Keywords:** General transit feed specification, indoor navigation, low-vision aid, mobile application, optical character recognition

## Abstract

Objective: People with blindness and low vision face substantial challenges when navigating both indoor and outdoor environments. While various solutions are available to facilitate travel to and from public transit hubs, there is a notable absence of solutions for navigating within transit hubs, often referred to as the “middle mile”. Although research pilots have explored the middle mile journey, no solutions exist at scale, leaving a critical gap for commuters with disabilities. In this paper, we proposed a novel mobile application, Commute Booster, that offers full trip planning and real-time guidance inside the station. Methods and procedures: Our system consists of two key components: the general transit feed specification (GTFS) and optical character recognition (OCR). The GTFS dataset generates a comprehensive list of wayfinding signage within subway stations that users will encounter during their intended journey. The OCR functionality enables users to identify relevant navigation signs in their immediate surroundings. By seamlessly integrating these two components, Commute Booster provides real-time feedback to users regarding the presence or absence of relevant navigation signs within the field of view of their phone camera during their journey. Results: As part of our technical validation process, we conducted tests at three subway stations in New York City. The sign detection achieved an impressive overall accuracy rate of 0.97. Additionally, the system exhibited a maximum detection range of 11 meters and supported an oblique angle of approximately 110 degrees for field of view detection. Conclusion: The Commute Booster mobile application relies on computer vision technology and does not require additional sensors or infrastructure. It holds tremendous promise in assisting individuals with blindness and low vision during their daily commutes. Clinical and Translational Impact Statement: Commute Booster translates the combination of OCR and GTFS into an assistive tool, which holds great promise for assisting people with blindness and low vision in their daily commute.

## I. Introduction

Metropolitan areas are home to about two-thirds of adults with disability in the United States [1]. Urban environments offer advantages such as improved socioeconomic status, and better access to public transportation, education, rehabilitation services, and healthcare, making them more desirable places to live for individuals with disabilities compared to suburban areas [1]. However, the accessibility of public transportation in urban areas remains an ongoing challenge for certain disability populations [2], [3]. A well-known issue is the so-called first mile/last mile problem, which is defined as the difficulty of commuters traveling from their origin to a transportation hub and vice versa. People with blindness and low vision (pBLV) encounter specific barriers during these initial and final segments of their journeys, such as the inadequate provision of information and obstructions on footpaths [2]. These barriers include, but are not limited to, inconsistent and discontinuous sidewalks, poorly maintained walkways, missing curb ramps, and insufficient crosswalks and crossing signals [4].

The middle mile problem, experienced by individuals with disabilities, refers to the challenging task of navigating within transportation hubs [3], [5], [6], [7]. Wayfinding in an unfamiliar environment often induces anxiety, leading to increased risks of getting lost and sustaining injuries [8]. While the availability of taxis and ride-sharing applications (apps) like Uber has reduced barriers, their high cost remains a limitation. A more affordable alternative is utilizing mass transit systems, such as subways. However, navigating a complex network of underground corridors, ticket booths, and platforms can be a daunting task, even for sighted individuals. While most public transportation hubs are compliant with the accessibility requirements of the Americans with Disabilities Act of 1990 (ADA) [9], pBLV still face tremendous challenges during travel. Although the ADA mandates certain provisions for sensory disabilities, such as rider information in large print, braille, or electronic formats in transportation hubs [10], pBLV require additional support to achieve full independence. For example, today, the majority of directional signage within the subway system is graphically illustrated or text-based. Also, braille signs are only posted on select columns inside the subway station. Visual search, which is difficult or impossible for many types of visual impairments [11], is a fundamental skill for locating text-based signs.

To address these travel challenges, we propose a mobile app called Commute Booster, providing pedestrians with detailed information covering every aspect of the planned route. This App supports full-trip planning and real-time travel support from the start location to the final destination, with detailed instructions every step of the way. The App can provide information to and from the station and support navigation within. Moreover, this App is modular and supports last-few-meters solutions, such as door detection near entrances at final destinations or object detection within areas of interest.

Many GPS-based apps support outdoor travel and are available at scale to facilitate first and last-mile journeys or travel to/from public transit hubs/access points, e.g., Google Maps [12], CityMapper [13], and Transit [14]. However, the navigation needs for the middle mile are still unmet. While research pilots have explored within-station navigation, no solution exists at scale [15], leaving a critical gap for commuters with sensory disabilities. In this paper, we focus on validating Commute Booster as a solution for the middle mile navigation problem.

Our new approach relies on general transit feed specification (GTFS), which is a data specification that allows public transit agencies to publish their transit data in a format that can be consumed by a wide variety of software apps [16]. The GTFS-pathways extension uses a graph representation to describe subway or train stations, with nodes representing locations and edges representing pathways [16]. The graph data structure fits into many existing path-planning algorithms so that we can enable path planning inside the subway station [17]. The proposed method can be scaled up to any public transportation system with basic GTFS and GTFS-pathways extension and with no need for additional infrastructure. We tested three subway stations in New York City (NYC) because the NYC subway system is known for its travel complexity [18].

The middle mile support component of Commute Booster employs optical character recognition (OCR) to detect navigation signs in the user’s vicinity and helps them determine if the detected sign is relevant. The middle mile support of Commute Booster requires basic user input and a phone with a camera. The user needs to specify the origin and destination. The phone camera enables the user to capture environmental images and provide real-time video frames as input to the App. The GTFS processing function takes the user input and retrieves relevant information about the journey of interest relative to GTFS files. This function returns a list of signs posted on this journey of interest. The OCR engine takes the video frame as the input and outputs text in the video frame with associated bounding boxes. A string similarity function determines if any OCR-detected text matches any item in the list of signs required to complete the journey of interest and prompts the user to follow the relevant sign.

The middle mile support pipeline of Commute Booster involves translating the combination of GTFS and OCR into an assistive app. Our experimental objective was to validate the performance and operation of this pipeline by conducting a series of experiments that focused on investigating detection accuracy, detection range, and the field of view detection angle.

## II. Related Work

Considering the prevalence of smartphones in the general public and in pBLV, mobile apps play important roles in activities of daily living, particularly during navigation [19]. Using the smartphone’s camera to capture information is now a tenable path forward.

One key example is NaviLens, a system consisting of vibrant and colorful QR-like codes that store venue information [20]. The core feature of this system is the camera of the smartphone, which reads codes while the user is moving [20]. These codes are strategically placed on existing signage, elevators, vending machines, restrooms, entrances, and other locations to convey pertinent transit details such as directions and public transit schedules. The app reads the information contained in a code out loud and provides train information in audio to help pBLV riders navigate the station. The field of view detection angle is stated to reach up to 160 degrees, and the largest codes can be detected from 60 feet away. However, the NaviLens’ approach requires a significant infrastructural investment, including the design, scripting, and then placement of codes. To provide a seamless navigation experience, the codes need to be posted at most, if not all, elevators, doors, and directional signs. Consequently, the coverage of NaviLens in NYC remains quite limited. Currently, the Metropolitan Transportation Authority (MTA) has tested NaviLens at one subway station (Jay St-MetroTech in downtown Brooklyn) and on one bus route (M23-SBS in Manhattan) through two pilot programs [21].

One widely used computer vision mobile app for pBLV is Seeing AI by Microsoft. Seeing AI uses the device camera to identify people and objects, which are then audibly described to the user [22]. Seeing AI is primarily used to describe short text, documents, products, people, currency, scenery, colors, handwriting, and light [22]. While Seeing AI can read navigation signs within subway stations, it encounters limitations in real public transportation hubs where there is an abundance of text, including advertisements, posters, maps, warning signs, and storefronts. It is extremely frustrating to read out all text and wait for relevant information. Also, it can be challenging for pBLV to extract useful navigation signage from the deluge of information. Therefore, Seeing AI may not serve as a practical navigation support tool in such contexts.

All Aboard is a mobile app developed by Massachusetts Eye and Ear that primarily focuses on aiding users in navigating the last few meters at public transit stops. It utilizes computer vision to detect bus stop signs in the vicinity of the user and guides the user to the precise location of the bus stop, providing distance estimations using computer vision algorithms [23]. For this app, there is no requirement for additional infrastructure, as it can recognize bus stops directly. The app notifies users about nearby bus stops and provides accurate distance information without overwhelming them with irrelevant details. However, while this functionality is beneficial for users with pBLV in utilizing bus services, their navigation needs in other transportation systems, such as subway stations, are still unaddressed.

Supervision Search is a mobile app specifically designed to assist pBLV in conducting visual searches. The app provides users with the convenience of inputting keywords through speech or typing, and then utilizes optical character recognition (OCR) to search for those keywords within images captured by the smartphone camera. When instances of the keyword are detected within the images, the app automatically zooms in on them, simplifying the retrieval of information related to the specific keyword [24]. Despite introducing an innovative framework that utilizes OCR to aid pBLV, the app does have a few limitations. One notable limitation is that users must manually enter keywords for each search, which can be cumbersome. Additionally, the app only supports keyword searches on still images and lacks real-time video functionality. This constraint significantly hampers the process of locating relevant information during navigation. While OCR shows promise as an approach, Thus, this app is not yet a fully functional navigation tool.

Another type of indoor navigation using smartphones involves utilizing constructed 3D models of indoor environments to facilitate indoor positioning and navigation. An application called ViNav is capable of generating 3D models of indoor spaces through the utilization of images sourced from the crowd. This app can also identify points of interest within these 3D models and create navigation paths for effective route planning [25]. ViNav employs image-based localization techniques to determine users’ positions and orientations, which helps calibrate the accuracy of trajectory calculations based on dead reckoning and sensor data collected during the user’s movement [25]. While this 3D modeling method demonstrates high precision, it faces challenges when scaling for use in public transportation systems. To cover the vast NYC subway system, a substantial amount of 3D modeling work would be required.

In summary, none of the navigation apps has completely resolved the challenge of in-station navigation. NaviLens utilizes QR-like codes to provide audio information for navigating transit stations but requires significant infrastructure investment. Seeing AI by Microsoft offers computer vision assistance but struggles with the abundance of text in real transit hubs. All Aboard aids users in navigating bus stops without additional infrastructure but does not address navigation needs in other transportation systems. Supervision Search assists in visual searches through OCR but requires manual keyword entry and lacks real-time video capabilities. ViNav proposes an accurate method for indoor navigation, but it requires a significant amount of 3D modeling work to cover the subway system.

GTFS is the other concept closely related to this paper and has demonstrated its value in various domains, including accessibility research, service level comparisons, and journey-planning apps [26]. For example, GTFS has been used to assess job accessibility and equity in public transportation across Canada [27]. Another research team developed PubtraVis, a tool that visualizes essential public transit operation characteristics such as mobility, speed, flow, and density using GTFS data [28]. Additionally, researchers created a mobile app that leverages GTFS to provide real-time bus arrival information, benefiting vulnerable road users accessing paratransit [29]. However, no previous study has explored the potential of utilizing GTFS for subway in-station navigation, indicating untapped opportunities within the GTFS dataset.

Our proposed app, Commute Booster, leverages GTFS to generate a comprehensive list of navigation signs users would encounter from their origin to destination within specific stations. By utilizing OCR technology, this App captures real-time environmental images and identifies relevant navigation signs in the user’s vicinity, providing navigation support for pBLV. Unlike NaviLens and ViNav, which require additional infrastructure, our app offers navigation support at all stations already mapped by the GTFS pathway projects, without the need for additional work. Furthermore, unlike Seeing AI, which reads out all captured texts, Commute Booster automatically determines the relevance of detected text and only conveys important navigation information to the user. With support for subway station navigation, Commute Booster facilitates navigation inside the subway station, which is commonly known as the main mode of transportation in many large cities, especially NYC. Commute Booster is an innovative integration of GTFS and OCR to assist pBLV in full trip support, inclusive of the middle mile.

## III. Methods and Procedures

**FIGURE 1.**
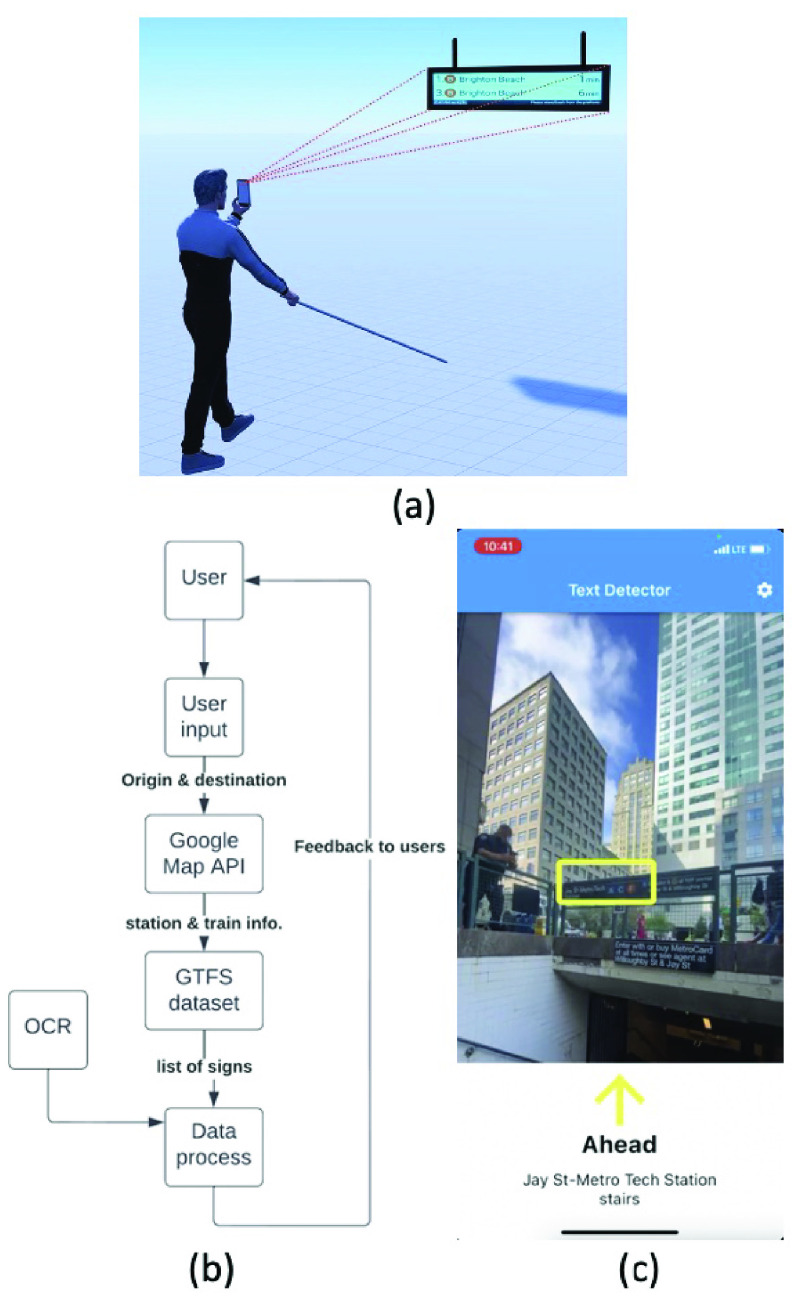
Schematics of Commute Booster. (a) Commute Booster inside a subway station. The user holds the phone, and the phone’s camera captures the surrounding environment in real-time. The App will inform the user if relevant signs are detected. (b) Workflow chart of Commute Booster. The user selects the origin and destination, as user input. The user input is fed into Google Maps API. Google Maps API returns trip information. The trip information is sent to the GTFS dataset. GTFS dataset returns a list of signs for the trip of interest. The data process stage compares the list of signs with the OCR results. If the relevant signs are detected, Commute Booster will provide haptic and verbal feedback to the user. (c) Screenshot of Commute Booster when a relevant sign is detected. When a sign is detected, the App highlights the location of the detected sign on the screen. At the bottom of this screen, it displays more detailed information (name of this sign, pathway modes, etc.) about this sign.

Commute Booster offers navigation support to and from public transit hubs and within these hubs. It utilizes the Google Maps API to provide navigation services in open areas. By integrating the Google Maps API into Commute Booster, the App is able to offer the same service as Google Maps. When a user arrives at a public transportation hub, the Google Maps API temporarily pauses, allowing the within-station navigation feature of Commute Booster to activate. Once the user exits the subway station, the within-station navigation feature concludes, and the Google Maps API service resumes. This paper specifically focuses on the within-station navigation aspect of Commute Booster, known as the middle mile. The schematics of the within-station navigation part of Commute Booster are shown in Figure 1.

The within-station navigation contains two main components: GTFS dataset processing and OCR result processing. The GTFS dataset processing generates a list of signs from the GTFS dataset. This list includes all signs that a user encounters from the entrance of interest to the platform of interest or vice versa if at the destination station. The OCR processing takes the real-time OCR reading results as input and determines if the reading result matches any item in the list of signs. The OCR result processing helps users to determine if the sign read by OCR is relevant and should be followed or not. We first describe the GTFS dataset processing in Section A, the OCR result processing in Section B, the string similarity processing in Section C, and feedback to users in Section D.

The GTFS dataset we adopted for this project is the GTFS-pathways project draft release which contains GTFS-pathways data for 106 stations and station 284 complexes, that was published by New York City Transit on May 26, 2020 [30].

### A. Processing of GTFS Dataset

As shown in Figure 1 (b), Commute Booster asks the user to specify origin and destination. Commute Booster uses the Google Maps location picker package [31], which enables the user to select places from maps directly without typing the actual coordinates. This package returns the user’s selection in the format of sets of location coordinates.

The coordinates of origin and destination are fed into Google Maps API. The Google Maps API is configured to find an optimal path by public transportation from origin to destination. By scanning the Google Maps API’s return value, Commute Booster obtains the name and the direction of the train of interest and the coordinates of stations for boarding or disembarking the train of interest.

#### 1) Finding the Id of the Entrance

**FIGURE 2.**
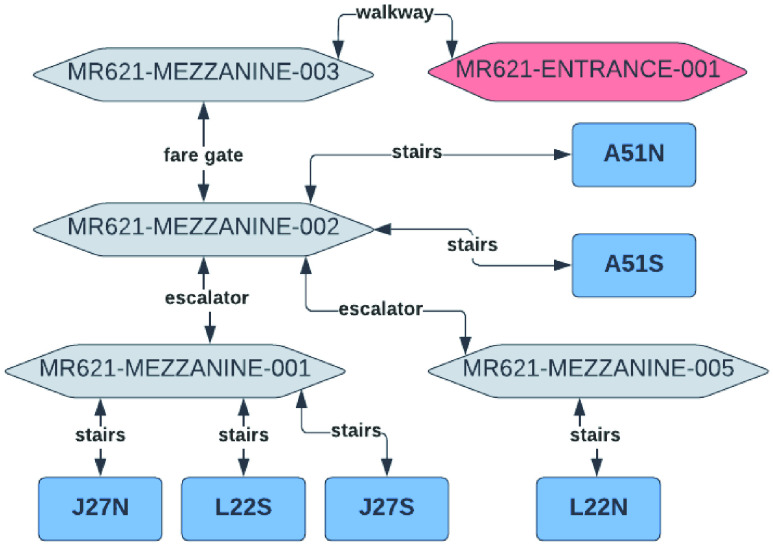
Visualization of the GTFS dataset for Broadway Junction Station (40.678919, −73.903453). The station ID is MR621. The red block represents a subway station entrance. The grey block represents a mezzanine. The blue block represents a platform. The text string on the block is the ID for that block. The lines between blocks represent pathways connection between blocks. The arrow on the line represents the direction of the pathway. The double-arrow line means this pathway is bidirectional.

As depicted in Figure 2, the GTFS dataset represents locations such as stations, platforms, entrances, mezzanines, etc., using unique IDs. These IDs are intended for internal use and are not readily understandable to the general public. In order to establish a connection between user-oriented Google Maps services and the GTFS dataset, it is necessary to convert the geographical coordinates obtained from the Google Maps API into location IDs.

One file in the GTFS dataset that can help with connecting Google Maps and GTFS is the GTFS-stops.txt. This file contains information such as IDs, coordinates, and more, pertaining to location-based stations. However, it is highly likely that the geographical coordinates provided by the Google Maps API slightly differ from the coordinates stored in the GTFS data, posing a challenge for achieving one-to-one matches between Google Maps and GTFS coordinates. To address this issue, Commute Booster utilizes the Haversine formula, a mathematical formula that can approximate the distance between two coordinates on the Earth [32]. The formula is defined as follows:

\begin{equation*} \scriptstyle D = 2R\arcsin \left({\sqrt {\sin ^{2}\left({\frac {\Delta \mathrm {lat}}{2}}\right) + \cos (\mathrm {lat1})\cos (\mathrm {lat2})\sin ^{2}\left({\frac {\Delta \mathrm {lon}}{2}}\right)} }\right)\end{equation*}

where lat1 is the latitude of the first point, and lat2 is the latitude of the second point. $\Delta $lat is the difference in latitudes, $\Delta $lon is the difference in longitudes, R is the radius of the earth, and D is the distance between points.

To determine the optimal entrance for accessing the subway station, Commute Booster employs the Haversine formula to calculate the distance between Google Maps’ suggested coordinates and each set of entrance coordinates in the GTFS dataset. This process enables Commute Booster to identify the entrance in GTFS that is closest to the suggested coordinates provided by Google Maps. The entrance associated with this minimal distance is considered the optimal location for entering the subway station. The corresponding ID for this entrance is the entrance ID for the particular trip. Additionally, for reference purposes, this particular entrance is referred to as the “entrance of interest,” and the station that includes this entrance ID is referred to as the “station of interest.”

#### 2) Finding the Id of the Platform

The platform is defined as the designated area alongside the rail tracks where passengers can board or alight from trains. In the GTFS dataset, platforms are also identified using unique IDs. It is common for subway stations in the NYC subway system to have multiple platforms. For example, the station depicted in Figure 2 consists of six platforms. Therefore, Commute Booster needs to determine the specific platform where the train of interest will stop. This platform is referred to as the “platform of interest” for reference.

To identify the platform of interest, the GTFS-stop-times.txt file within the GTFS dataset comes in handy. This file contains information regarding the schedules for train arrivals and departures at various platforms for each trip. The trip ID serves as a unique identifier for specifying train trip schedules. If Commute Booster can ascertain the trip ID associated with the desired trip, it can determine the platforms that the train of interest will visit. The trip ID itself contains details about the train’s name and direction, which can be used to locate the corresponding trip ID.

**FIGURE 3.**
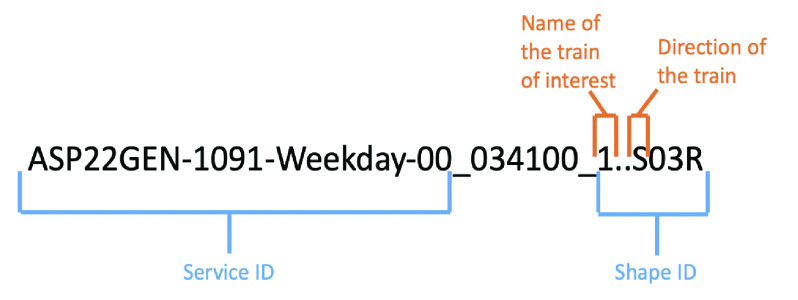
Example of the trip ID. The trip ID always ends with the shape ID. The shape ID contains the name of the train and the direction of the train. Notice that the direction of the train is either N (North) or S (South), which represents that the train is heading toward the north terminal or south terminal. The Google Maps API returns the direction as the head sign. The head sign can be converted to N or S according to [33]. The service ID can be figured out by determining whether the time of the planned trip is a weekday or a weekend. By matching the train name and the train direction between IDs and Google Maps’ suggested values, Commute Booster can retrieve the ID for the trip of interest.

The Google Maps API provides information on the name and direction of the train of interest. By comparing the name and direction of the train obtained from the Google Maps API with the trip IDs listed in the GTFS-stop-times.txt file, Commute Booster can identify the trip ID of interest. The process of matching is illustrated in Figure 3. Once the trip ID is determined, Commute Booster can extract all the platforms associated with that trip ID. These platforms are then sorted based on the departure time of trains from each platform. The sorted platforms are stored in a list known as the “list-platform-train,” which represents the order of platforms that the train of interest will visit during its trip.

**FIGURE 4.**
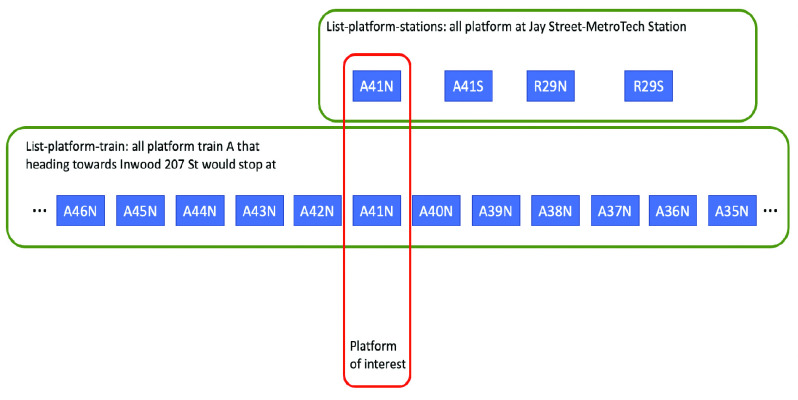
Example of determining the platform of interest from the list-platform-train and the list-platform-station. The list-platform-train represents the sequence of platforms that A line (8 Avenue express) toward Inwood 207 Street would stop at. The list-platform-station represents all platforms at Jay St-MetroTech Station. By comparing these two lists, Commute Booster can figure out which platform at Jay St-MetroTech Station that the A line would stop at, which is platform A41N.

The list-platform-train is not equivalent to the platform of interest. Here, the platform of interest means the platform ID that the user needs to visit to travel on to the train of interest. To figure out the platform of interest, the App determines which ID in the list-platform-train also exists in the station of interest. By scanning the GTFS-stops.txt, Commute Booster selects platforms that share the same name as the station of interest. In the current naming convention, the platform’s name is the same as the name of the station the platform belongs to. Commute Booster filters out the platforms that are more than 1 km away from the entrance of interest to handle stations in different boroughs but have the same names. Commute Booster selects all qualified platforms and stores them in a list called list-platform-station. Therefore, as shown in Figure 4, there must exist one and only one platform that exists in both the list-platform-train and the list-platform-station. This platform is the platform of interest for this trip.

#### 3) Path Planning

This App adopts the classical Dijkstra algorithm [34], which finds the shortest path between nodes for path planning in subway stations. To use Dijkstra’s algorithm, this App first converts the subway stations’ floor plans into a graph data structure. A GTFS file that can help with this is the GTFS-pathways.txt. This file uses a graph representation to describe subway stations, with nodes representing locations, including entrances, mezzanines, platforms, etc., and edges representing pathways [35]. The graph of the station of interest is constructed by retrieving all relevant entries from the GTFS-pathways.txt.

It is necessary to specify the origin and the destination. For entering the subway station, the origin would be the entrance ID, and the destination would be the platform ID. For exiting the subway station, the origin would be the platform ID, and the destination would be the entrance ID. The algorithm returns a list that represents the smallest number of nodes needed to visit in order to travel from the origin to the destination. This list is referred to as the list-of-nodes.

#### 4) Generating the List of Signs

The list-of-nodes itself is not enough for navigation. It is necessary to convert the list into some useful navigation information. Therefore, Commute Booster searches all signs posted between every two items in the list-of-nodes. By finding two nearby items in the list to the GTFS-pathways.txt, Commute Booster identifies pathways between two locations. Commute Booster retrieves posted signs associated with that pathway and stores these posted signs into the list of list-of-signs.

### B. OCR

The list-of-signs is fundamental for navigation, but Commute Booster needs to ensure this information is accessible for pBLV. Therefore, this App captures all texts in the surrounding environment around the user and alerts the user if relevant navigation signs are detected.

#### 1) Google Ml Kit OCR

Commute Booster utilizes Text Recognition from Google ML Kit for OCR [36]. A previous study has shown that Google ML Kit is the most robust OCR engine for keyword search in urban environments [24]. Google ML Kit can process input images in real-time, detect text regions, recognize characters, and group them into words.

### C. OCR Error Handling

OCR reading results in subway stations are greatly influenced by factors such as illumination conditions, image quality, and distance. Due to the inevitable occurrence of OCR reading errors, post-processing techniques are commonly employed to enhance the accuracy of the readings. Here, we present a post-processing algorithm specifically designed to process OCR reading results within the NYC subway system.

#### 1) Dice Coefficient

A string similarity metric is essential for assessing how similar or alike two strings are. Given the presence of OCR errors, this App does not mandate an exact match between the OCR-detected text and the actual sign. In this paper, Commute Booster utilizes the Dice coefficient, a statistical measure that quantifies the similarity between two sets of data [37], namely,

\begin{equation*}\mathrm {Coefficient} = 2 \frac {|A \cap B|}{|A| + |B|}\end{equation*}

where A represents the first string and B is the second string.

#### 2) Raw OCR Reading

In practice, if the Dice coefficient exceed the threshold of 0.7, Commute Booster can confidently conclude that the OCR-detected sign matches the ground-truth sign. For instance, the initial OCR reading for the sign “Jay Street-Metro Tech Station” is “RJaySt-MetroTechACStationCE”. Given that the Dice coefficient between the ground truth sign text and the OCR reading text is 0.85, it is reasonable to conclude that these two texts are identical.

However, this approach encounters challenges within the NYC subway system due to the presence of highly similar signs. For example, the sign “Jay St & Willoughby St” and the sign “Fulton St & Willoughby St” represent completely distinct locations. but the Dice coefficient between these two signs is 0.8, indicating these two signs are identical based on the Dice coefficient threshold of 0.7.

#### 3) Error Correction

Commute Booster proposes an algorithm to handle the issue of highly similar signs. This algorithm contains three steps, which are defined as follows:

(i) Generate a dictionary of signs in NYC: Firstly, this algorithm extracts every item from the sign-posted-as and reversed-sign-posted-as fields in the GTFS-pathways.txt. These items are stored in a list. Theoretically, this list contains every sign posted in the NYC subway system. This list of all signs is referred to as the dictionary of signs. Once generated, this list is retained in the App. This step will be skipped if such a list already exists in the App.
(ii) Determine if a subway sign is detected: This algorithm calculates the Dice coefficient between the OCR reading texts and every item in the list obtained in step (i). The item with the highest Dice coefficient to the OCR reading texts is considered a candidate. For this step, we set the threshold for the Dice coefficient to 0.7. If the calculated coefficient is greater than or equal to this threshold, Commute Booster recognizes that a subway sign is detected. This detected sign is referred to as the candidate for reference purposes. However, this candidate is not necessarily relevant to the planned trip. The algorithm will proceed to step (iii) to determine its relevance. If the calculated coefficient is smaller than this threshold, Commute Booster concludes that the OCR reading text string is not a subway sign, and the algorithm stops at this point.
(iii) Determine the relevance: This algorithm calculates the Dice coefficient between the candidate and every item in the list of signs (list-of-signs). Please note that the list-of-signs is the collection of signs the user will encounter along the desired path. The threshold of the Dice coefficient is set to 0.85. If the candidate is in the list-of-signs, there should be a single item within that list with a Dice coefficient of 1. Nevertheless, we establish the threshold at 0.85 to allow for a certain level of error tolerance in this process. If the Dice coefficient between the candidate and any item in the list-of-signs is greater than the threshold, Commute Booster determines that a relevant sign has been detected. Otherwise, Commute Booster concludes that a MTA sign detected but this sign is irrelevant to current trip.

**FIGURE 5.**
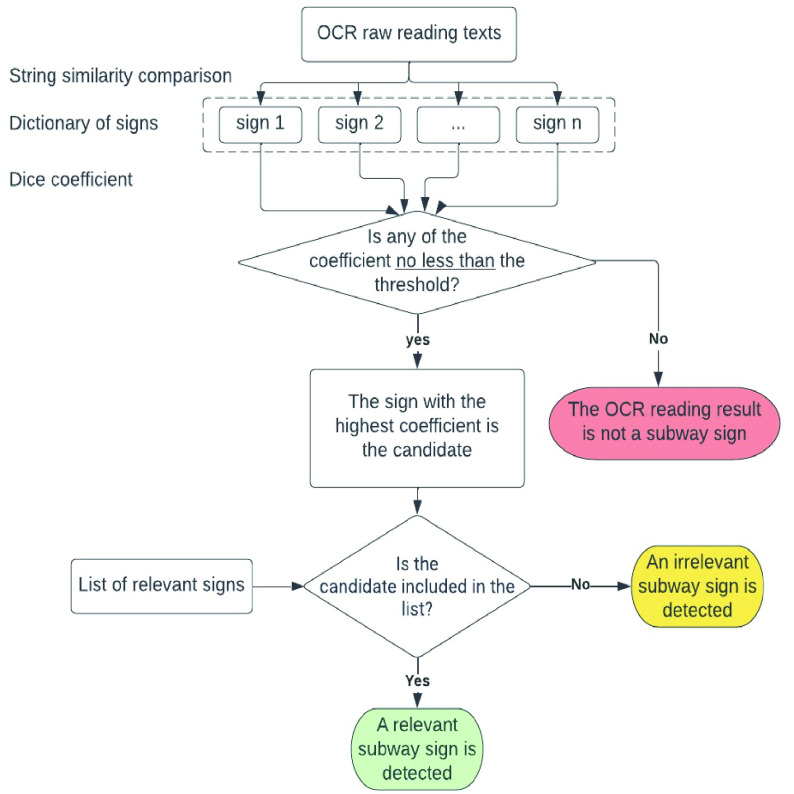
Flow chart of proposed sign detection algorithm. Firstly, the algorithm compares the OCR reading text string with a dictionary of signs, evaluating the string similarity coefficient. If none of the coefficients exceed 0.7, the algorithm determines that the OCR reading string does not correspond to a subway sign. Conversely, if there is at least one coefficient greater than 0.7, the algorithm selects the sign from the dictionary with the highest coefficient as a candidate. If this candidate is in the list of relevant signs, the algorithm concludes that a relevant subway sign has been detected. However, if the candidate is not found in the list of relevant signs, the algorithm determines that an irrelevant subway sign has been detected.

This algorithm repeats itself every time the OCR component reads a new text string. The flow chart of this proposed method is shown in Figure 5.

This method can handle extremely similar signs. Consider the example “Exit — Jay St & Willoughby St” (sign A) and “Exit — Fulton St & Willoughby St” (sign B). The most similar item in the subway sign dictionary to sign A is the sign A itself, which has a Dice coefficient of 1.00, and the next most similar sign in this system is sign B, with a Dice coefficient of 0.78. Thus, only sign A is considered as the candidate, and sign B is discarded. Similar signs are successfully differentiated.

Previous studies [38], [39], [40] confirmed the reliability of utilizing string similarity comparison and candidate ranking algorithms for OCR error correction. However, these studies primarily focus on general-purpose OCR error correction, relying heavily on contextual information for error detection and language corpus for correction. In the case of navigation signs, where contextual information is often lacking, these OCR correction methods cannot be directly applied. Our algorithm draws inspiration from the principles of string similarity comparison and candidate ranking algorithms for error correction, but it is specifically tailored to the context of subway signs. Section IV provides compelling evidence of the high accuracy achieved by our proposed method.

### D. Feedback to Users

Once Commute Booster detects relevant signs, it will alert users with both haptic and verbal feedback. The mobile phone continuously vibrates as long as a relevant sign is detected. The verbal feedback employs text-to-speech technology to inform the user that a sign is detected. If an MTA posted navigation sign is detected, but this sign is not relevant to the user’s trip, Commute Booster will alert users to do not follow this sign. If only non sign-related texts are detected, Commute Booster will keep playing a sonar-like sound to let the user know Commute Booster is searching, but no sign is detected. The user should keep moving around in this case.

## IV. Experiments

We carried out two experiments to evaluate the technical validity of Commute Booster. The first experiment evaluated the accuracy of this App, and the second evaluated the detection range and field of view detection for relevant signage. We describe these experiments and their results separately in the following sections.

### A. Experiment 1: Accuracy

#### 1) Setup

Within the actual subway station environment, a wide range of situational text is present. This text encompasses various types such as navigational signs, warning signs, store names, door number plates, advertisements, posters, and graffiti, among others. As the proposed App leverages OCR, it is important to take into account different sources of text in the actual environment of interest.

**FIGURE 6.**
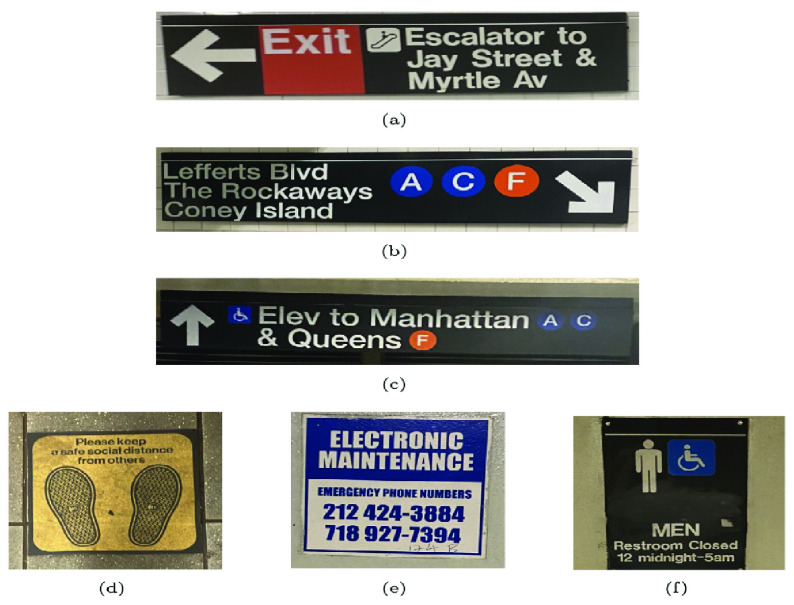
Examples of images captured: (a-c) are examples of sign images, and (d-f) are examples of environmental images.

For this experiment, we captured photographs of all discernible text within three real subway stations: Jay St-MetroTech Station (40.6907721, −73.9952596), Dekalb Avenue Station (40.6907721, −73.9952596), and Canal Street Station (40.7123858, −74.011083). To identify signs posted by the MTA, we employed the definitions outlined in the MTA Sign Manual [41]. Any images containing text not specified in the MTA sign manual were categorized as environmental text. We made the best efforts to capture all instances of environmental text within these specific subway stations. The photographs were taken using the iPhone 11 ultra-wide-angle camera, maintaining a consistent distance of 3 to 5 meters and ensuring a fronto-parallel plane (head-on) perspective. In this experiment, images featuring identical text were only considered once. Examples of these images are depicted in Figure 6.

These images form three experimental datasets. The first dataset contains 160 images captured at Jay Street-MetroTech station (Jay), including 58 images of MTA signs. The second dataset contains 78 images captured at Dekalb Avenue Station (Dekalb), including 34 images of MTA signs. The third dataset contains 142 images captured at Canal Street Station (Canal), which includes 40 images of MTA signs.

We designed eight trials at Jay, four trials at Dekalb, and four trials at Canal. A trial tests a path from a street-level entrance to a platform or vice versa. The trial number, trial origin, and trial destination are in Table 1.

**TABLE 1:** Experimental Results at Jay Street-MetroTech Station, Dekalb Avenue Station, and Canal Street Station

(a) Trials at Jay Street-MetroTech Station.							
Trial	Origin	Destination	TP	TN	FP	FN	Total
1	Entrance-010	A41N	3	152	4	1	160
2	A41N	Entrance-010	3	156	1	0	160
3	Entrance-003	A41S	3	154	2	1	160
4	A41S	Entrance-003	3	156	1	0	160
5	Entrance-011	R29S	3	153	2	2	160
6	R29S	Entrance-011	3	156	1	0	160
7	Entrance-013	R29N	3	153	2	2	160
8	R29N	Entrance-013	3	156	1	0	160
Avg.			3	154.5	1.75	0.75	160

(b) Trials at Dekalb Avenue Station.							
Trial	Origin	Destination	TP	TN	FP	FN	Total
1	Entrance-001	R30N	3	72	2	1	78
2	R30N	Entrance-001	3	73	2	0	78
3	Entrance-004	R30S	3	72	2	1	78
4	R30S	Entrance-004	3	73	2	0	78
Avg.			3	72.50	2.00	0.50	78

(c) Trials at Canal Street Station.							
Trial	Origin	Destination	TP	TN	FP	FN	Total
1	Entrance-003	A34N	4	136	0	2	142
2	A34N	Entrance-003	4	136	2	0	142
3	Entrance-005	A34S	4	136	0	2	142
4	A34S	Entrance-005	4	136	2	0	142
Avg.			4	136.0	1.00	1.00	142

The dataset of images taken from these subway stations consists entirely of station-pertinent text, making them sufficient to simulate the actual station environment. We installed Commute Booster on an iPhone 11 and displayed these images on a computer monitor. For each trial, we manually designated the origin and destination. The details of the origin and destination for each trial are displayed in Table 1. Each image was displayed for a maximum of 5 seconds to ensure efficient processing. Commute Booster’s OCR and algorithms are specifically designed for real-time processing, achieving a practical speed of approximately 20 frames per second on the iPhone 11. While Commute Booster can recognize text with minimal latency, we have set a threshold of 5 seconds. As a result, we conclude that Commute Booster fails to recognize a sign only if it is displayed for a minimum of 5 seconds and does not provide a correct response.

The mobile device is 20± 1 cm away from the center line of the computer monitor. Both the monitor and the mobile device are vertical to the ground. The computer monitor used for this experiment is a 16.2-inch display with a 3456-by-2234 native resolution of 254 pixels per inch. The brightness is set to 1000 nits. The color space for this monitor is DCI-P3, and the refresh rate for this monitor is set to 60 Hz. To standardize the size of the text in images, we adjust the zoom-in level of each image so that the initial letter for the string in an image is 0.7± 0.2 cm in height. Notice that this operation of letter standardization would destroy the distance information. In this experiment, we exclusively examine the accuracy of OCR detection. We will discuss the distance-related information in experiment 2.

In each dataset, the images are arranged in the following order: street-level images, mezzanine-level images, and platform-level images. Within each level, the images are organized chronologically based on their timestamp. For trials starting from an entrance and heading towards a platform, these images will be presented to Commute Booster in this specific order. Conversely, for trials starting from a platform and heading towards an exit, the images will be displayed in the reversed order. To be considered correctly identified, a sign must satisfy two criteria: the text string must match, and the level must correspond. In other words, if the text on a street-level sign matches the text on a sign from a mezzanine-level or platform-level image, it will still be considered a misidentified sign.

We conducted this experiment using various smart devices, such as the iPhone 11 (iOS), third-generation iPad Pro (iPad OS), and OnePlus 8 (Android). When a device has a single rear camera, Commute Booster automatically selects it as the default camera. For devices with dual-camera systems (wide and ultra-wide) or triple camera systems (wide, ultra-wide, and telephoto), Commute Booster automatically chooses the ultra-wide camera. The processing time for sign detection varies slightly across different devices but does not affect the detection results. Any smart device with similar specifications, such as a 10MP ultra-wide camera with a 120-degree field of view plus auto-focus and auto-exposure capabilities, can achieve similar results. While image quality can impact OCR detection, as mentioned in the Google ML Kit Guides [36], the camera specifications of the top 10 best-selling smartphones in 2022 [42] ensure that they can all deliver comparable or superior results. In fact, Commute Booster is designed to seamlessly operate on any mobile system and we invested significant effort in ensuring its robustness during the application development phase.

The trial is completed once all images from that dataset are shown. We measured the number of images that Commute Booster correctly identified, misidentified, and ignored.

#### 2) Results

Table 1 summarizes the experimental results of datasets for Jay, Dekalb, and Canal. In this table, true positives (TP) represent the number of signs relevant to the planned path that Commute Booster predicted as relevant. True negatives (TN) represent the number of signs that are irrelevant to the planned path, and Commute Booster predicted as irrelevant. False positives (FP) represent the number of signs that are irrelevant to the planned path, but Commute Booster predicted as relevant. False Negatives (FN) represent the number of signs that are relevant to the planned path, but Commute Booster predicted as irrelevant.

We report the accuracy, precision, and F1 scores of Jay, Dekalb, and Canal stations, respectively. The F1 score, a popular performance metric for classification, is calculated as the harmonic mean of precision and recall [43]. Table 2 and Table 3 show these measurements. Commute Booster achieved an average accuracy of 0.9777 with a precision of 0.6401 and an F1 score of 0.7128 for all trials. The details of these misidentified signs are analyzed in the Discussion section. Commute Booster is able to accurately distinguish navigation text in complex environments.

**TABLE 2:** Performance Measurements for Trials Jay Street-MetroTech Station, Dekalb Avenue Station, and Canal Street Station

(a) Performance measurements for trials in Table 1(a).			
Trial	Accuracy	Precision	F1 Score
1	0.9688	0.4286	0.5455
2	0.9938	0.7500	0.8571
3	0.9813	0.6000	0.6667
4	0.9938	0.7500	0.8571
5	0.9750	0.6000	0.6000
6	0.9938	0.7500	0.8571
7	0.9750	0.6000	0.6000
8	0.9938	0.7500	0.8571
Avg.	0.9844	0.6536	0.7300

(b) Performance measurements for trials in Table 1(b).			
Trial	Accuracy	Precision	F1 Score
1	0.9615	0.6000	0.6667
2	0.9744	0.6000	0.7500
3	0.9615	0.6000	0.6667
4	0.9744	0.6000	0.7500
Avg.	0.9697	0.6000	0.7084

(c) Performance measurements for trials in Table 1(c).			
Trial	Accuracy	Precision	F1 Score
1	0.9859	0.6667	0.8000
2	0.9859	0.6667	0.8000
3	0.9859	0.6667	0.8000
4	0.9789	0.6667	0.8000
Avg.	0.9789	0.6667	0.7000

**TABLE 3:** Performance Measurements of Commute Booster at Jay, Dekalb, and Canal, Respectively

Location	Accuracy	Precision	F1 Score
Jay	0.9844	0.6536	0.7300
Dekalb	0.9697	0.6000	0.7084
Canal	0.9789	0.6667	0.7000
Average	0.9777	0.6401	0.7128

### B. Experiment 2: Range

#### 1) Setup

The purpose of this experiment is to assess the detection field of view and range for signage. The experimenter chose a navigation sign that Commute Booster could accurately recognize (a true positive sign). Holding the mobile phone, the experimenter positioned themselves 1 meter away from the sign and moved along a 1-meter plane until this App could no longer read the sign. The points at which Commute Booster began to fail were noted by the experimenter. This process was then repeated at increasing distances of 1, 2, 3, 4, and so on, meters away from the sign, with each trial incrementing the distance by 1 meter until the sign became unreadable. These steps were replicated for 10 different signs. The graphical representations of this process can be observed as the blue lines in Figure 9 and Figure 10.

**FIGURE 7.**
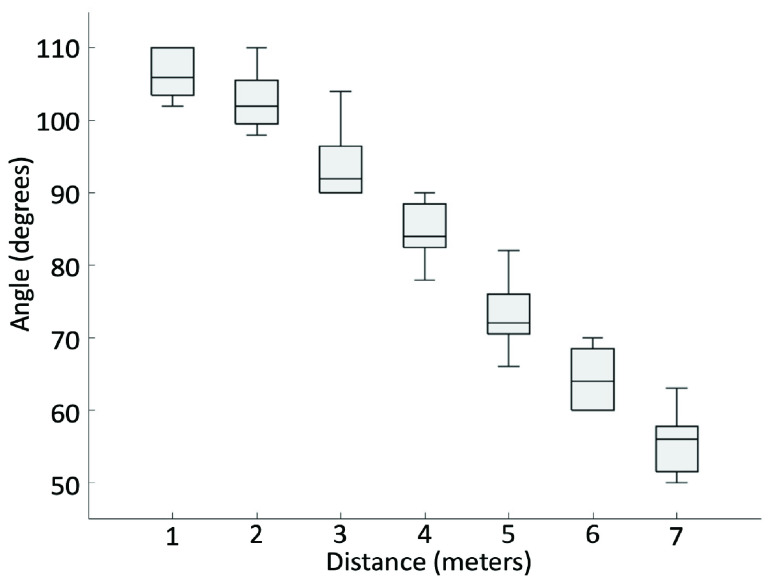
Boxplot graph showing the relationship between distance and angle for a within-station sign. The horizontal axis represents the distance from posted signs to the camera. The vertical axis represents the maximum angles that signs are readable.

**FIGURE 8.**
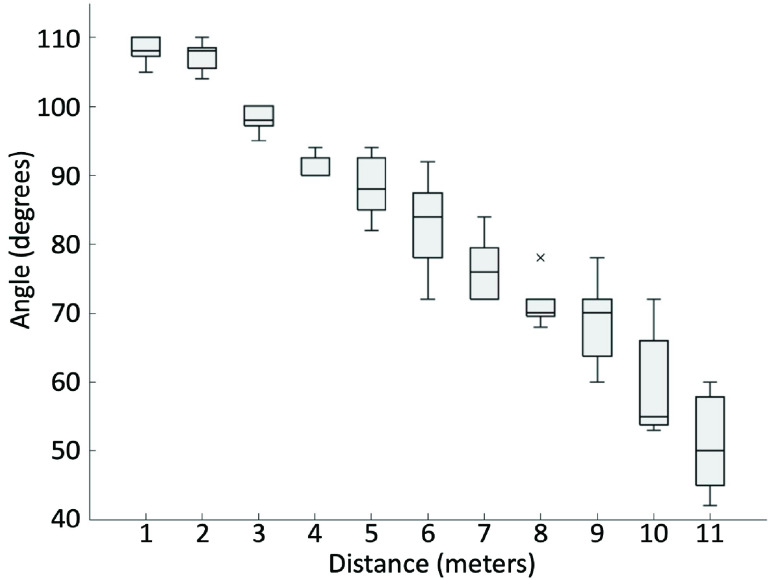
Boxplot graph showing the relationship between distance and angle for a street-level sign. The horizontal axis represents the distance from posted signs to the camera. The vertical axis represents the maximum angles that signs are readable.

**FIGURE 9.**
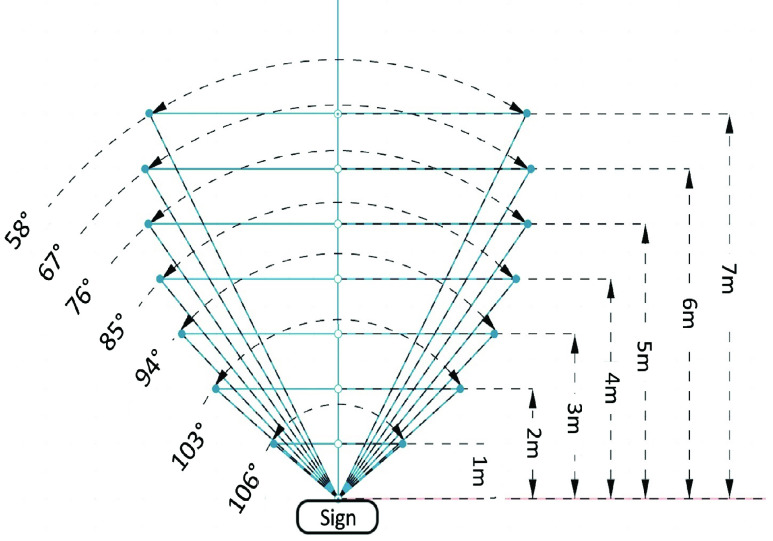
Schematic representation of the change in angle depending on the distance between the sign to the smartphone for a within-station sign. The distance values shown on the right side of the plot represent the distance from the sign to the camera. The angle values shown on the left side of the plot represent the average maximum field of view detection angles that signs are readable at a specific distance. The area enclosed by the blue lines is the field of view detection.

**FIGURE 10.**
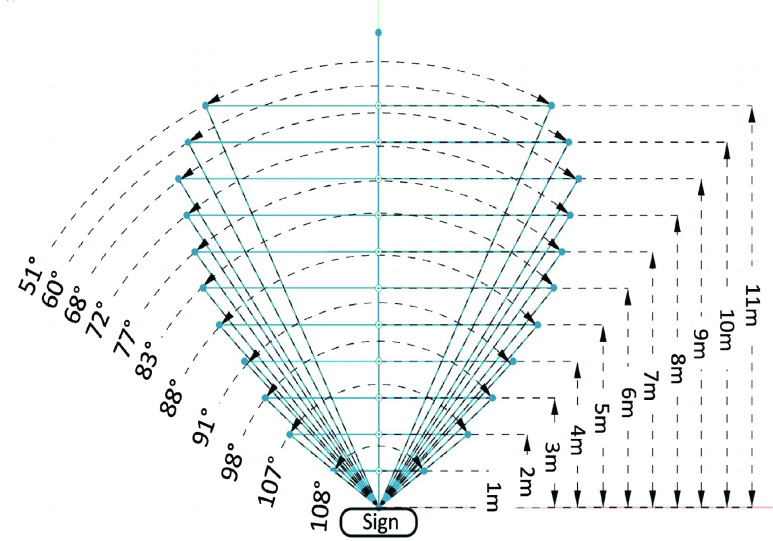
Schematic representation of the change in angle depending on the distance between the sign to the smartphone for a street-level sign. The distance values shown on the right side of the plot represent the distance from the sign to the camera. The angle values shown on the left side of the plot represent the average maximum field of view detection angles that signs are readable at a specific distance. The area enclosed by the blue lines is the field of view detection.

#### 2) Results

We conducted measurements on 10 different signs to determine their distance and field of view detection angles. Half of these signs were directional signs placed within the subway station, while the other half were station name signs positioned at street level. The result of the within-station directional signs is shown in Figure 7. The schematic representation of the change in angle depending on the distance is shown in Figure 9. Based on these two figures, we can conclude that within-station signs can be read from a distance of up to 7 meters, with a detection angle of up to 106 degrees. Similarly, Figure 8 shows results for the street-level signs. The schematic representation of the change in angle depending on the distance is shown in Figure 10. From these two figures, it is evident that street-level signs can be read up to 11 meters away, with a detection angle of up to 110 degrees.

Based on Figure 7 and Figure 8, we conclude that there exists a negative correlation between the angle and the distance. As the distance from the sign increases, the angle of detection becomes narrower. These two figures provide clear evidence that Commute Booster is capable of reading signs across a considerable range. Perfect alignment between the sign and the phone’s camera is not necessary. This range is sufficient for navigating within subway stations, considering that many areas within the stations are relatively narrow. The experimental results are promising and serve to validate the performance of the underlying approach.

## V. Discussion

Compared to the within-station sign, which is often posted in a relatively narrow indoor environment and used at close range, the street-level sign is expected to be recognized at a farther distance. Street-level signs often have better illuminating conditions, especially during the daytime, the font size is larger in general, and it is posted in a more spacious environment. Indeed, the experimental results verify these assertions, as street-level signs can be recognized up to 11 meters away. This is significantly higher than the within-station sign that can be read only at 7 meters, at maximum.

From the experimental results for classification, it is reasonable to conclude the accuracy of Commute Booster is acceptable, with an accuracy of 0.978, precision of 0.640, and an F1 score of 0.713. However, as an App developed for the pBLV, the reliability needs to be perfect. The precision still needs to be improved as several types of images are unable to be handled perfectly by Commute Booster. In section A below, we will discuss several types of images that negatively affected the performance of Commute Booster. In section B, we will discuss the applicability of Commute Booster. In section C, we will discuss the future work.

### A. Type of Errors

#### 1) Purely Graphical Navigation Sign

**FIGURE 11.**
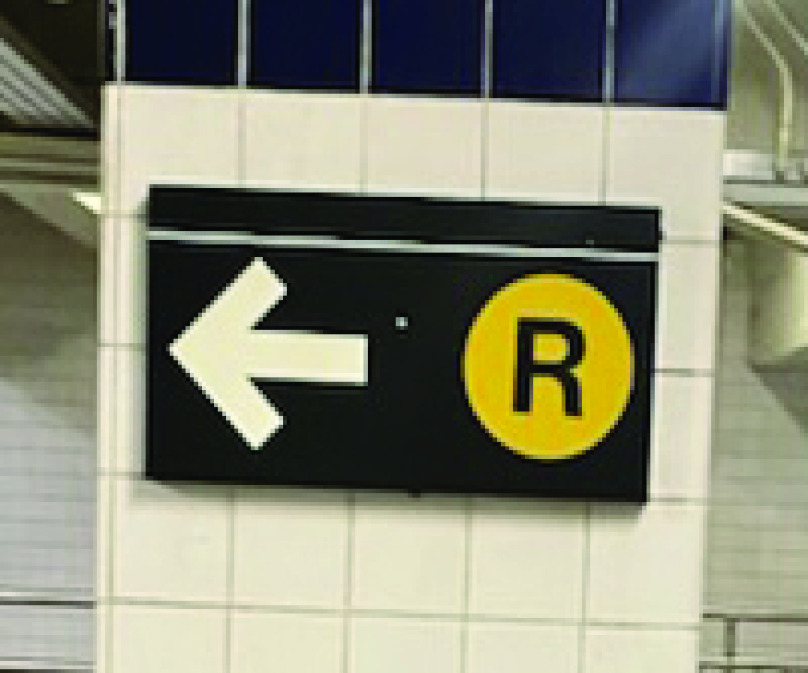
Example of a purely graphical-based sign.

In the MTA navigation sign system, some navigation signs are purely graphical based, as shown in Figure 11. The information on this type of navigation sign is not stored in the GTFS dataset. Also, in the example of Figure 11, there is one character in this type of sign. Even if the OCR engine only misrecognized one character, the whole sign would not be correctly recognized. Commute Booster has a difficult time processing purely graphical navigation signs. We are considering computer vision algorithms in future development to solve the issue of the graphical-based signs.

#### 2) Misclassified Environmental Texts

**FIGURE 12.**
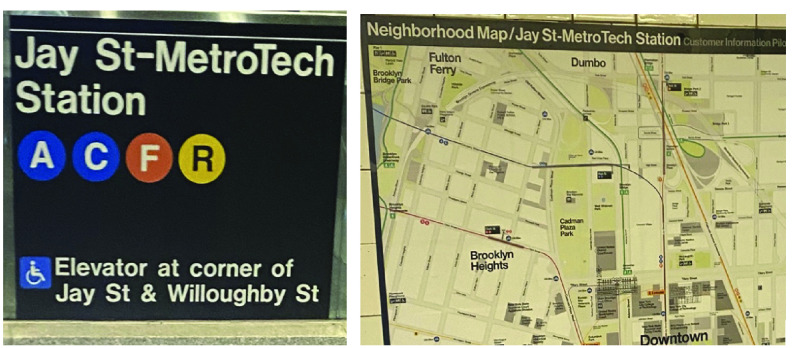
Example of the environmental misclassified environmental texts. The image on the left is a street-level entrance subway sign. The image on the right is a map posted inside the subway station titled Neighborhood Map/Jay St-MetroTech Station.

There is environmental text that occasionally interferes with the app’s detection. Commute Booster sometimes mistakenly identifies unrelated environmental texts as navigation signs. As shown in Figure 12, the image on the left displays the subway station navigation sign with the text “Jay St-MetroTech Station”. On the right, there is a neighborhood map that is also titled “Jay St-MetroTech Station”. However, Commute Booster mistakenly suggested the neighborhood map as a navigation sign. In future development, we plan to implement an object detection algorithm capable of recognizing navigation signs, ensuring that the OCR only includes text relevant to navigation signs.

#### 3) GTFS Pathway Missing Data

The GTFS pathway project is an ongoing initiative. In the current version of the NYC subway GTFS, not all pathways are documented. There are certain stations that have no recorded pathways, while others have incomplete records. Additionally, some crucial information, including the sign-posted-as and reversed-sign-posted-as fields, is missing in certain stations in the current NYC subway GTFS. The absence of this GTFS-pathways data can potentially lead to inaccurate path planning. However, as the GTFS-pathways project progresses, this issue is expected to be alleviated. It is worth noting that other cities, like Boston [44], possess nearly complete datasets. In such cities, Commute Booster may exhibit enhanced performance due to the availability of comprehensive data.

### B. Applicability of Commute Booster

GTFS is the most widely used format for specifying public transit schedules and currently has data from 1,327 providers in 677 locations worldwide [45]. Theoretically, as a navigation App that relies solely on GTFS and OCR, Commute Booster can be applied to any transit stations covered by GTFS. However, for full functionality, Commute Booster requires the presence of GTFS-pathways. GTFS-pathways is an extension that has been incorporated into the GTFS specification in recent years (March 2019) [35], resulting in very limited coverage for transit systems. We hope that highlighting the value of GTFS-pathways in this research will motivate public transit providers to expedite the construction of GTFS-pathways extension and encourage researchers to pursue further translational research connecting GTFS with assistive technology.

### C. Future Work

As an App developed for pBLV, it is essential to conduct human subjects research to evaluate the potential benefits of Commute Booster for daily commuting. Following this technical validation, our first clinical testing, under development, aims to recruit both participants with blindness/low vision and participants with normal vision. To assess for efficacy, participants will be asked to use a mobile App to navigate along an optimal route from pre-selected subway station entrances to designated platforms and vice versa; several trials will be performed. The study will be designed as a randomized crossover with participants being randomly assigned to either the Commute Booster then Google Maps app or Google Map then Commute Booster app. Google Maps is our comparator due to its dominant position in the mobile navigation app market [46] and its inclusion of basic in-station navigation support. Performance metrics, including travel duration, distance, speed, number of collisions/falls, number of directional losses, and subjective feedback, will be measured for each participant. We hypothesize that Commute Booster will enhance the navigation experiences of both cohorts, objectively and subjectively, namely in efficiency, i.e., time taken, and distance traveled. We will recruit participants with diverse visual pathologies, such as peripheral vision loss, central vision loss, and/or acuity loss. The severity of vision loss will range from mild to no light perception.

## VI. Conclusion

In this paper, we present a novel mobile app called Commute Booster for pBLV. This App utilizes the smartphone’s camera to capture real-time images and isolate navigation signs in the user’s surrounding environment, based on their specified origin and destination. Commute Booster employs the Google Maps API to generate an optimal route connecting the origin and destination.

This App leverages GTFS datasets to decipher relevant signs along the user’s journey from the station entrance to the subway platform and from the subway platform to the exit. It uses OCR technology to continuously capture environmental images and determine the relevance of text, providing real-time navigation support.

Our experiments aimed to validate the technical feasibility of this approach. In the first experiment, we utilized this App to scan hundreds of images with text from three different subway stations. Commute Booster accurately selected relevant navigation signs from the environmental images, with an average accuracy of 0.97 and an average precision of 0.64. The second experiment tested the maximum detection field of view/range for sign recognition. The results showed that signs within the station could be read up to 7 meters away, with a detection field of view of almost 110 degrees. Street-level signs could be read up to 11 meters away, with a field of view of almost 110 degrees. The angle and distance were found to be negatively correlated, meaning that signs could be recognized at wider angles when the user moved closer to them.

This App showcases the integration of GTFS and OCR technologies into a comprehensive navigation solution for pBLV. The conducted experiments provide compelling evidence that validates the feasibility of this innovative approach. As a pioneering exploration of a novel technological modality, this study falls within the realm of basic research in the field of translational science.

This App operates entirely through vision-based technology and does not require additional sensors or infrastructure. It can run on any smartphone, holding great promise in assisting pBLV individuals during their daily commutes. Future research will focus on employing more advanced computer vision algorithms to recognize navigation signs, with the aim of reducing false positives and improving overall performance. Additionally, as part of our translational engineering study, we plan to conduct a human subject study in the near future to further validate the performance of Commute Booster.
